# The influence of quality maternity waiting homes on utilization of facilities for delivery in rural Zambia

**DOI:** 10.1186/s12978-017-0328-z

**Published:** 2017-05-30

**Authors:** Elizabeth G. Henry, Katherine Semrau, Davidson H. Hamer, Taryn Vian, Mary Nambao, Kaluba Mataka, Nancy A. Scott

**Affiliations:** 1 Department of Global Health, Boston University School of Public Health, Boston, USA; 2 Ariadne Labs, Boston, USA; 3 Division of Global Health Equity, Brigham & Women’s Hospital, Boston, USA; 4 Department of Medicine, Harvard Medical School, Boston, USA; 5 Section of Infectious Diseases, Department of Medicine, Boston University School of Medicine, Boston, USA; 6 Zambia Center for Applied Health Research and Development (ZCAHRD) Limited, Lusaka, Zambia; 7 Ministry of Health, Lusaka, Zambia

**Keywords:** Maternity waiting home, Facility-based delivery, Maternal health, Newborn health, Skilled birth attendance, Zambia

## Abstract

**Background:**

Residential accommodation for expectant mothers adjacent to health facilities, known as maternity waiting homes (MWH), is an intervention designed to improve access to skilled deliveries in low-income countries like Zambia where the maternal mortality ratio is estimated at 398 deaths per 100,000 live births. Our study aimed to assess the relationship between MWH quality and the likelihood of facility delivery in Kalomo and Choma Districts in Southern Province, Zambia.

**Methods:**

We systematically assessed and inventoried the functional capacity of all existing MWH using a quantitative facility survey and photographs of the structures. We calculated a composite score and used multivariate regression to quantify MWH quality and its association with the likelihood of facility delivery using household survey data collected on delivery location in Kalomo and Choma Districts from 2011–2013.

**Results:**

MWH were generally in poor condition and composite scores varied widely, with a median score of 28.0 and ranging from 12 to 66 out of a possible 75 points. Of the 17,200 total deliveries captured from 2011–2013 in 40 study catchment area facilities, a higher proportion occurred in facilities where there was either a MWH or the health facility provided space for pregnant waiting mothers compared to those with no accommodations (60.7% versus 55.9%, *p* <0.001). After controlling for confounders including implementation of *Saving Mothers Giving Life*, a large-scale maternal health systems strengthening program, among women whose catchment area facilities had an MWH, those women with MWHs in their catchment area that were rated medium or high quality had a 95% increase in the odds of facility delivery than those whose catchment area MWHs were of poor quality (OR: 1.95, 95% CI 1.76, 2.16).

**Conclusions:**

Improving both the availability and the quality of MWH represents a potentially useful strategy to increasing facility delivery in rural Zambia.

**Trial registration:**

The Zambia Chlorhexidine Application Trial is registered at Clinical Trials.gov (identifier: NCT01241318)

## Plain English summary

Most maternal deaths could be averted with improved access to skilled care and facilities equipped to handle obstetric emergencies. In Zambia, where only 56% of rural women deliver in a facility, cost and distance are critical barriers to accessing care. Maternity waiting homes (MWH) have been proposed to address the distance problem. The purpose of this study was to determine if women who have access to MWH are more likely to deliver at a facility, and if the quality of the MWH matters.

Data sources from two separate studies conducted in Southern Province, Zambia, between 2011 and 2013 included survey data from 17,200 pregnant women enrolled at their first antenatal care visit and followed through delivery, and both health facility and MWH assessments. The woman-level data included background characteristics, collected during the enrollment survey, and self-reported location of delivery, collected during a household survey after delivery. The health facility and MWH assessments included indicators of capacity and quality. Statistical methods were used to examine the relationship between utilization of facilities for delivery and MWH quality.

In our study, women whose catchment area health facilities had an MWH or a designed waiting space had higher rates of facility delivery. Moreover, the higher the quality of the MWH, the more likely a woman was to deliver at a facility, regardless of the facility’s capacity to address obstetric emergencies. MWH are a potential solution to the distance problem and should be considered as one possible intervention to improve access to facility delivery in Zambia.

## Background

An estimated 62% of global maternal deaths occur in sub-Saharan Africa, where a woman’s lifetime risk of maternal death is 1 in 59, far higher than the risk in all low-income countries, estimated at 1 in 160 [1]. More than 80% of maternal deaths are due to direct causes associated with obstetric complications [2] and could be prevented with the provision of timely and appropriate intrapartum care. The World Health Organization (WHO) has recommended skilled care at every birth, which includes having a skilled attendant (someone trained to manage normal pregnancies and to identify, manage and refer complications) present for the birth as well as access to facilities with the capacity for emergency obstetric care [3]. In Zambia, the lifetime risk of maternal death is 1 in 38 [1], and just about half of women living in rural areas of the country (56%) deliver at facilities, compared to 89% in urban settings [4]. As with other low-income countries, Zambia continues to face persistent challenges to implementing the WHO skilled birth attendance recommendation.

The factors contributing to the delays in seeking, reaching and accessing quality maternal care, per the Three Delay model, are well established. Key factors affecting utilization and health outcomes include household-level illness recognition and awareness of obstetric complications; women’s status, education level, and other socioeconomic factors; perceived accessibility of health care facilities and perceived quality of care that the woman would receive; community-level transportation and referral challenges, and whether a facility actually has adequate infrastructure and clinician capacity to recognize and address clinical needs [5, 6]. Costs, transport and distance to the facility, which lead to the second delay of reaching care, have been repeatedly identified as key drivers of the low utilization of facilities for maternity care in Zambia [7–10]. While the government has abolished user fees for maternal and child services to increase financial access to health care services in 2006, about 65% of the population lives in rural areas and still face physical barriers to access. A recent analysis of the effects of the abolished user-fee policy in Zambia suggests that this reform has not overcome key barriers to utilization of public sector facilities for delivery, and that both quality of care and difficulties related to distance may be a more important determinants [11]. Additional evidence confirms that the odds of facility delivery in rural Zambia decreases as distance to a facility increases [12].

Maternity waiting homes (MWHs), residential lodging near a health facility, represent a potential strategy to improve accessibility and utilization of facilities for delivery. The WHO has recommended MWHs as an intervention to improve maternity care [13]. Women who might otherwise not have access to skilled care due to the constraints posed by distance could benefit by staying at a MWH and being closer to a facility that can manage emergency obstetric complications. MWHs of some sort have been implemented since the beginning of the 20^th^ century in more than 18 countries around the world, including the United States, Canada and Northern Europe, Cuba, India and several countries in sub-Saharan Africa including Zimbabwe, Nigeria, Uganda, Ethiopia and Malawi [14, 15]. However, there is mixed evidence of the effectiveness of MWH on both utilization of health facilities for maternity care and health outcomes [16].

With governments eager to find feasible solutions to the distance problem, there is a resurgence of interest in constructing MWH and developing policies around their implementation. However, it is not yet clear if this will be a worthwhile investment among competing priorities. Evidence from Zimbabwe suggests that when a facility has an MWH women are much more likely to deliver there [17]. The perceived quality of the MWH, among other factors such as direct and indirect costs of staying at the MWH, may also play a role in rates of MWH utilization, including in rural Zambia [15, 18]. However, there is no evidence on whether or not the actual quality of the MWH is associated with utilization of facilities for delivery.

In Zambia, women are more likely to deliver in facilities better equipped to handle obstetric emergencies, independent of distance to the facility, suggesting that quality of care is also an important factor in decision making about facility-based birth [12]. It is important, therefore, to also understand whether the relationship between MWH quality and facility utilization would remain regardless of the quality of the health facility in terms of capacity to deal with obstetric emergencies. It is possible that the quality of the health facility is the key driver of facility delivery, rather than the quality of the MWH. The purpose of our study was two-fold: to determine whether the existence of an MWH at a facility would predict women’s utilization of the facility for delivery, and to determine whether the quality level of the MWH would predict the magnitude of this relationship, adjusting for facility quality to handle obstetric emergencies.

## Methods

### Study setting

Our study utilized data collected from two separate studies conducted in two contiguous districts in Southern Province, Kalomo and Choma Districts, between 2011 and 2013: 1) a formative evaluation of MWH conducted in 2013 on the physical quality of MWHs [19]; and 2) a cluster-randomized controlled trial conducted in Southern Province between 2011 and 2013 [20].

At the time of data collection, Kalomo had a primarily rural population (93%) of 258,570 [21] and 35 health facilities, including 27 health centers (HC), six health posts and two referral hospitals [22]. Choma District also had a mostly rural (76%) population of 247,860 [21] with 29 HCs, six health posts and two referral hospitals. We would most likely expect to find an MWH at the HCs and hospitals, but not health posts, as they do not provide delivery services. Of those hospitals and HCs that offer delivery services, at the time of our survey 25 of Kalomo facilities had MWHs (86%), whereas only six in Choma had MWHs (19%) [22].

In Kalomo District during the time of our study, there was an ongoing initiative to improve maternal health by addressing the three delays through a public-private partnership called *Saving Mothers, Giving Life (SMGL)* [23–25]*.* The package of interventions implemented through SMGL since 2012 have included, among others, community mobilization and sensitization activities, improvements in referral systems, mentoring health staff, and investments in supply chain and equipment at facilities. There is evidence that SMGL had a significant impact on rates of facility delivery in Kalomo [26]. Therefore, the effect of this package of interventions is addressed in our analysis as a potential confounder.

### Data sources

Data on facility delivery were obtained from the Zambia Chlorhexidine Application Trial (ZamCAT), a cluster Randomized Controlled Trial (RCT) in which 39,797 pregnant women in six districts of Southern Province, including Choma and Kalomo, were enrolled at their first antenatal care (ANC) visit and followed through 28 days post-delivery [20, 27]. The goal of ZamCAT was to evaluate the effectiveness of using chlorhexidine cord cleansing to reduce neonatal mortality. The woman-level data included background characteristics, collected during the initial enrollment survey, and location of their delivery, collected during a survey after delivery at the 1 and 4 day postpartum household visits.

Data for facility capacity for emergency obstetric care were obtained from a health facility assessment (HFA) tool conducted as part of ZamCAT between June and August 2013. Not all facilities in each of the two districts were included in the ZamCAT study. Facility-level criteria for inclusion in the study were: (1) an estimated 160 births per year in the catchment area, (2) routine provision of ANC services, and (3) willingness to participate. In Kalomo District, 22 facilities, all HC, were selected, representing 81% of all HC in the district and in Choma 18 facilities (HC) were selected, representing 62% of all HC in the district. The HFA tool captured basic indictors of capacity to perform maternal and newborn health signal functions and other indicators of routine maternity and newborn care. Signal functions are a set of medical interventions that address the direct causes of maternal death [28]. Full details of the ZamCAT trial are described elsewhere [20, 27].

### Indicators

In this analysis, the main outcome indicator was delivery at any facility, determined by location of birth (health facility or hospital) reported by the mother at the ZamCAT household postpartum visit. Indicators of woman’s socio-economic status (household wealth, education level), maternal demographics (age, marital status), and pregnancy characteristics (parity, ANC) were obtained from the ZamCAT enrollment questionnaire.

The indicator for facility capacity for emergency obstetric care was a continuous score, calculated as the sum of the basic emergency obstetric and newborn care (EmONC) signal functions (maximum of 7) [28] and 1 point for each of the following: electricity, water, 24-h care, and availability of a skilled provider (defined by the WHO as someone trained to manage normal pregnancies and to identify, manage and refer complications) [3], for a total 11 possible points. Signal functions were assessed by asking the facility in-charge interviewee whether or not the function had been performed within the last 3 months at that facility.

The primary independent variable was a composite quality score for the MWH. As part of a formative evaluation [19], the study team systematically assessed and inventoried the functional capacity of all existing MWHs in the selected districts (*n* = 31; 25 in Kalomo and 6 in Choma). The health facilities with existing MWHs were identified in advance by the district health offices. The composite quality score was created by utilizing both quantitative and qualitative data collected at each MWH, inclusive of a series of questions asked of a member of affiliated clinic staff as well as photos that captured the state of the physical structure/space, availability of a water source within 200 m, availability/state of a toilet and bedding, and availability/state of a gathering and cooking area. These items emerged from our literature review of the physical factors that may be important determinants of quality in an MWH. In seven cases, facilities did not have a separate MWH structure at the time of assessment, but instead utilized a clinic area as a designated space for waiting women. We evaluated these spaces using the same criteria.

The scoring system for each criterion ranged from 0 (not available) to 5 (present and fully functional). There were a total possible 75 points if all criteria for each of the 15 components was rated highest**.** Based on their composite score, MWHs were also categorized into tertiles and labeled as “low”, “medium” and “high” quality.

#### Analysis

We limited our analysis to those catchment areas for which we had health facility and woman-level data from ZamCAT (*n* = 40 sites; 18 of these had an affiliated MWH, three had a designated area for waiting mothers, often the clinic wards, and 19 of these had no structure nor designated area). Our sample of sites excluded seven health facilities in Kalomo and three in Choma that had an MWH but for which we did not have ZamCAT woman-level data. Of these 10 excluded, three facilities were hospitals, as hospital catchment areas defined differently than for HCs, and thus were not assigned for randomization for ZamCAT. The other non-hospital health facilities/HCs were not included in the ZamCAT study because they did not meet the health facility inclusion criteria.

We conducted bivariate analyses examining associations between background characteristics of the women in our sample and our outcome of facility delivery to determine what factors to control for in the regression analyses. We used the Pearson chi-square test for categorical variables and t-tests for continuous variables if the data were normally distributed or non-parametric Wilcoxon rank-sum tests if non-normally distributed. Any characteristics associated with outcome variables with *p*-value <0.20 were included in the adjusted logistic regression model.

We used multiple logistic regression to assess the likelihood of facility delivery based first on the facility capacity score and then on the composite MWH quality score. We also regressed the primary individual-level outcome (facility delivery) against the category of the MWH quality (low, medium, high), adjusting for covariates that may have moderated the effect, such as sociodemographic characteristics and distance to the facility, as well as for facility capacity score and level of SMGL program implementation. SMGL level of implementation was defined by using three time periods: data on women who 1) delivered before or during January 2012, the time at which the SMGL rollout started; 2) women who delivered between February and August 2012 and may have had some exposure to the SMGL program; and 3) women who delivered from September 2012 to the end of the ZamCAT data collection in October 2013 and were most likely exposed to some level of SMGL activities. In Choma the SMGL implementation level was always zero as SMGL never operated in that district during those time periods. Quantitative data were analysed in SAS version 9.3 [29].

## Results

### Sample characteristics

A total of 17,200 women were included in the final analysis. Women had a median age 25.0 (IQR 20.0-31.0), 85.4% were married and more than 91% had at least some primary education (Table 1). Just over 22% had no children prior to this pregnancy, and 37% reported living more than two hours from a health facility (regardless of type of transportation used). Just over 5% of women were recorded as HIV positive on their ANC cards. More than half of women in our sample (58.5%) delivered at any facility (*n* = 10,069). Woman’s age, marital status, education level, parity, ANC visits, HIV status and distance to facility were all associated with facility delivery. Older women, women who were married, had fewer years of education, more than one child, lived more than two hours away from a health facility, had less than the four recommended ANC visits, and were not HIV positive were less likely to deliver in a facility. Just over half of women (55%) lived in catchment areas with an existing MWH or designated waiting space for pregnant mothers.

**Table 1:** Characteristics of the women with and without facility delivery and unadjusted odds of facility delivery

Characteristic	Sample distribution *n* = 17,200 (%)	Delivered in a facility *N* = 10,069 (%)	Delivered at home *N* = 7131 (%)	Unadjusted odds of delivering in a facility
Woman’s age in years (%)				
15–19	22.4	25.6	17.8	1.0
20–24	27.5	26.6	28.8	0.64 (0.59, 0.70)***
25–34	36.3	34.6	38.8	0.62 (0.57, 0.67)***
35–49	13.8	13.3	14.5	0.64 (0.57, 0.71)***
Age in years (median, IQR)	25.0 (20.0-31.0)	24.0 (19.0-30.0)	25.0 (21.0-31.0)	
Household size (median, IQR)	6.0 (4.0-8.0)	6.0 (4–8)	6.0 (4–8)	
Currently married (%)				
No	14.6	17.4	10.6	1.0
Yes	85.4	82.6	89.4	0.56 (0.51, 0.62)***
Mother’s highest education (%)				
None (0)	8.3	7.0	10.2	1.0
Any primary (1–7)	55.0	50.0	62.1	1.17 (1.05, 1.31)**
More than primary (7+)	36.7	43.0	27.7	2.27 (2.02, 2.55)***
Asset quartile (%)				
First/poorest	26.6	25.7	27.7	1.0
Second	24.5	22.9	26.9	0.92 (0.84, 0.99)*
Third	24.7	24.8	24.6	1.09 (0.99, 1.18)
Fourth/highest	24.2	26.6	20.8	1.38 (1.27, 1.51)***
Parity (%)				
0	22.3	27.7	15.6	1.0
1	17.7	17.5	18.1	0.51 (0.46, 0.56)***
> = 2	60.0	54.8	67.4	0.43 (0.39, 0.46)***
Distance to health facility ≥ 2 h (%)**^a^				
No	62.7	69.1	53.6	1.0
Yes	37.3	30.9	46.4	0.52 (0.49, 0.55)***
4 ANC visits (%)				
No	55.0	48.7	63.9	1.0
Yes	45.0	51.3	36.1	1.87 (1.75, 1.99)***
Mother HIV positive (%)				
No	94.4	92.8	96.8	1.0
Yes	5.6	7.2	3.2	2.34 (2.00, 2.74)***

^a^Self-reported time from home to nearest health facility

*p <0.05, **p <0.01, ***p <0.001

Characteristics of health facilities, both those with and without affiliated MWHs, as well as those that accommodate pregnant women by designating some space for waiting, are included in Table 2. On the 11-point scale for the composite score, facility scores ranged from 2 points to 9 points with a mean of 5.8 (SD 1.8). Facilities with an MWH, as well as those that provide space for waiting pregnant women, appeared to differ systematically from those that did not have a MWH, with those with an MWH having a higher overall score of 6.8 (SD 1.5) compared to those without (4.8 (SD 1.6)). Nearly 60% of facilities with an MWH had performed four or more signal functions, compared to only 16% of those without a MWH. A much higher proportion of facilities with a MWH had administered antibiotics for maternal infections in the past three months (100.0% versus 42.1%). This trend was the same for nearly all signal functions, though not always as pronounced. None of the facilities without a MWH had electricity, whereas seven facilities with a MWH did. Nearly all facilities had access to clean water, provided obstetric services 24/7 and, except for two facilities with a MWH, had at least one skilled provider trained in deliveries on staff. On average those facilities with an MWH had a higher average number of deliveries per month (22.4 (SD 17.2)) than in those without an MWH (17.2 (SD 15.0)). Facilities with a designated waiting space had the highest average number of deliveries, with a mean of 25.0 (SD 14.0).

**Table 2:** Characteristics of health facilities, with and without Maternity Waiting Homes (MWH)^a^

Facility characteristics	All study facilities	Facilities have an MWH		
	N(%)	None n(%)	Yes n (%)	Designated Area for Waiting Mothers n (%)
	40 (100.0)	19 (100.0)	18 (100.0)	3 (100.0)
Signal functions				
Four or more signal functions	15 (37.5)	3 (15.8)	11 (61.1)	1 (33.3)
Parenteral antibiotics for maternal infection	27 (67.5)	8 (42.1)	18 (100.0)	1 (33.3)
Parenteral oxytocic drugs for hemorrhage	38 (95.0)	18 (94.7)	17 (94.4)	3 (100.0)
Parenteral magnesium sulfate for eclampsia	9 (22.5)	3 (15.7)	4 (22.2)	2 (96.7)
Manual removal of the placenta	10 (25.0)	3 (15.7)	6 (33.3)	1 (33.3)
Removal of retained products of conception	10 (25.0)	2 (10.5)	7 (38.9)	1 (33.3)
Assisted vaginal delivery	4 (10.0)	0 (0.0)	4 (22.2)	0 (0.0)
Resuscitation with bag and mask of non-breathing baby	24 (60.0)	7 (36.8)	15 (83.3)	2 (66.7)
Infrastructure and human resources				
Electricity	8 (20.0)	0 (0.00)	7 (38.9)	1 (33.3)
Water supply	37 (92.5)	17 (89.5)	17 (94.4)	3 (100.0)
Service availability 24/7	36 (95.0)	15 (79.0)	18 (100.0)	3 (100.0)
At least 1 skilled provider	38 (95.0)	19 (100.0)	16 (88.9)	3 (100.0)
Total composite score				
Mean (SD)	5.8 (1.8)	4.8 (1.6)	6.8 (1.5)	6.3 (2.5)
Median (Range)	6.0 (2.0-9.0)	5.0 (2.0-8.0)	7.0 (4.0-9.0)	6.0 (4.0-9.0)
Average number of deliveries (monthly)	20.1 (15.9)	17.2 (15.0)	22.4 (17.2)	25.0 (14.0)

^**a**^Those with no MWH but with designated areas for waiting mothers are presented separately because they are distinctly different from those with a MWH and from those with no MWH

MWHs in our study were generally in poor condition. All MWHs had some type of water supply, toilet, and sanitation inside and outside the shelter. Nearly all of the MWHs had some type of floor, roof and general structural integrity (Table 3). However, most did not have beds or mattresses, nor separate bathing areas for women. We observed wide variation in quality between MWHs, with composite scores ranging from 12 to 66 out of a possible 75, and a median of 28.5 with an inter-quartile range (IQR) of 23–41.

**Table 3:** Characteristics of 18 existing Maternity Waiting Homes (WH), Kalomo and Choma Districts

MWH characteristics	Number (%) Any present *N* = 18	Score Mean (SD)	Score Median (IQR)
Beds	2 (11.1)	0.6 (1.6)	0.0 (0–0)
Mattresses	3 (16.7)	0.7 (1.7)	0.0 (0–0)
Electricity	6 (33.3)	1.4 (2.1)	0.0 (0–4)
Sanitation inside	18 (100.0)	3.1 (1.1)	3.0 (2–4)
Sanitation outside	18 (100.0)	2.8 (1.1)	2.5 (2–3)
Security	18 (100.0)	3.1 (1.6)	3.0 (1–5)
Water supply	18 (100.0)	2.9 (0.8)	3.0 (2–3)
Toilet	18 (100.0)	2.6 (0.9)	3.0 (2–3)
Community support	6 (33.3)	0.7 (1.1)	0.0 (0–2)
Structure integrity	18 (100.0)	3.2 (1.2)	3.0 (2–4)
Roof	18 (100.0)	1.0 (0)	1.0 (2–5)
Floor surface	18 (100.0)	4.2 (1.5)	5.0 (2–5)
Shower/bathing area	5 (27.8)	0.7 (1.2)	0.0 (0–0)
Cooking area	18 (100.0)	2.2 (1.3)	2.0 (1–3)
Total score	NA	32.7 (13.9)	28.5 (23–41)

### Relationship between maternity home quality and facility delivery

The rate of facility delivery for women whose catchment area had any MWH (including a designated space for waiting) was 60.7 (5698/9385), compared with 55.9 (4371/7815) for those without any MWH or waiting space, an absolute difference of 4.7 points. Adjusting for maternal age, any maternal education, household asset quartile, parity, maternal HIV status, four or more ANC visits, distance to health facility, and whether or not the SMGL program was operational in the district when the mother went for delivery (but not facility capacity for emergency response), there was a 37% relative increase in the odds of facility delivery for women living in catchment areas with an MWH or designated space, compared to women whose catchment areas did not have any MWH (OR: 1.37, 95% CI: 1.27, 1.46) (results not shown in table). Additionally, we tested for any effect the ZamCAT intervention may have had on facility delivery rates. The unadjusted relationship between women living in ZamCAT intervention areas, as compared to non-intervention areas, and facility delivery was non-significant (*p* = 0.08, OR: 0.947, 95% CI: 0.89, 1.01) (results not shown in table) and therefore was not included in the adjusted model.

Our results also indicate that facility capacity for emergency response has an independent effect on utilization of facilities for delivery. As quality of the facility increases, women are more likely to delivery there (1.10, 95% CI: 1.08, 1.12) (results not shown). This relationship is still significant when we adjust for confounders (maternal age, any maternal education, household asset quartile, parity, maternal HIV status, four or more ANC visits, distance to health facility, and whether or not the SMGL program was operational in the district when the mother went for delivery) (1.10, 95% CI: 1.08, 1.12). When we then revisited the relationship between having an MWH (or designated space) and facility delivery but adjusted for facility capacity, as well as the covariates listed above, the odds of facility delivery was still 19% higher for those with any MWH or accommodation than those without (1.19, 95% CI: 1.10, 1.29) (Table 4).

**Table 4:** Likelihood of facility-based birth by Maternity Home: any and quality level

	No. of Births	Facility-based Births No (%)	Odds ratio (95% CI)	Adjusted OR (95% CI)
MWH or Waiting Space at Facility				
No^a^	7815	4371 (55.9)	1.00	1.00
Yes	9385	5698 (60.7)	1.22 (1.15, 1.29)	1.19 (1.10, 1.29)
Type of Waiting Space				
None^a^	7815	4371 (55.9)	1.00	1.00
Space but no MWH	1392	1055 (75.8)	2.47 (2.17, 2.81)	2.13 (1.85, 2.46)
MWH	7993	4643 (58.1)	1.09 (1.03, 1.16)	1.07 (0.98, 1.16)
Rank of MWH^c^				
Low^a^	3142	1550 (49.3)	1.00	1.00
Medium/High	4851	3093 (63.8)	1.81 (1.65, 1.98)	1.95 (1.76, 2.16)

^a^reference group

^b^Controlling for maternal age, any maternal education, asset quartile, parity, maternal HIV status, four or more ANC visits, distance to health facility, SMGL program implementation, facility capacity

^c^Only among those with an existing MWH structure and a score (missing 3 facilities with designated spaces that were not able to be scored)

When we differentiate between facilities that had an actual MWH structure and those that had only designated accommodations for pregnant waiting mothers, such as space in the wards, as compared to those without any place for waiting, there is a less pronounced positive relationship between facility delivery and MWH among those with just a structure (OR: 1.09, 95% CI:1.03, 1.16), but a large increase in the odds of facility delivery among those women whose catchment areas provided space but no formal MWH structure (OR:2.47, 95% CI: 2.17, 2.81). Adjusting for the same covariates as above, the odds of facility delivery becomes non-significant for those with just an MWH, compared to those with none (1.07, 95% CI: 0.98, 1.16) but the effect is still positive for those with a designated space (OR: 2.13, 95% CI: 1.85, 2.46).

When we examined the level of quality of the MWH, only among those that were scored (excluding those with waiting spaces as they did not have the same criteria upon which they could be measured), the rate of facility delivery in those with a low quality MWH was 49.3%. This was lower than the rate of facility delivery in catchment areas with a medium or high-level quality MWH (63.8%). When we examined the relationship between MWH quality and facility delivery, again limiting our analysis only to those facilities with existing MWH structures and controlling for those covariates listed above, including facility capacity score, there was a 95% relative increase in the odds of facility delivery for high or medium-ranking MWHs compared to low-ranking shelters, adjusting for covariates (OR: 1.95, 95% CI: 1.76,2.16).

## Discussion

Women in our study were more likely to deliver at health facilities when their catchment area health facility had an MWH or a designated space for waiting on the premises than those women with no immediate access to an MWH or waiting space. When controlling for facility capacity to handle obstetric emergencies, the relationship between presence of an MWH or designated space for waiting and utilization of facility for delivery remained. This is consistent with findings from previous work in Zambia that showed that the absence of a suitable shelter for expecting mothers was a contributing factor to low utilization of maternal health services [30] and a study in Liberia where availability of MWHs decreased the distance barrier for women to access skilled care for childbirth [31].

The finding that those women with a designated space had a much higher odds of facility delivery than those with no MWH or space suggests that there may be a characteristics about the facility, such as perceived supportive staff that are willing to accommodate waiting women when no space is formally available, that may influence women’s decisions to deliver there. Women’s cultural beliefs and perceptions of facility-based care have been found to be major drivers of facility delivery [32]. In Tanzania, a woman’s previous or current experience with the health system, including whether or not she was treated with respect during ANC or delivery, was found to be more important that the perceived quality of the facility itself [33]. Our study findings indicate there is something unique about facilities that provide waiting space for mothers, and this warrants further investigation.

In our study the quality of the MWH is an important determinant of utilization. Among those facilities that had an MWH structure, women were more likely to deliver at facilities if the structures were of higher quality. The low-quality MWH had about the same utilization rates as those with no MWH at all. This suggests that if MWH are constructed in the future, particular attention should be paid to both the inclusion and the quality of components such as proper bedding, private spaces for bathing, and areas for cooking, among others. These findings are consistent with results from a review of MWH utilization which cited several factors that influence a woman’s decision to stay in an MWH, including the small size of the home and the lack of proper hygiene of the facilities [16]. In our region of Zambia, the poor state of the MWHs, including inadequate sleeping spaces and bedding, water and sanitary services, have been cited as factors that deterred women from utilizing MWH [18]. Additional research from Zambia has illuminated that poor conditions of the MWH led some husbands to forbid their wives from using them [34]. Community acceptance and support have been shown to be critical factors in the successful introduction of MWHs in Ethiopia [35]. Our findings provide additional evidence that considering the needs of the community will be integral to the potential success of the utilization of MWH in Zambia.

There are several limitations to this analysis. First, although we have the self-reported type of facility at which a woman delivered, we were not able to determine the actual delivery location of the woman, whether at her health facility catchment area (HFCA), or a neighboring facility or hospital. Our study is assuming that most women delivered in their catchment area’s facility, and would have been influenced by the presence or absence of an MWH in their HFCA. It is possible that women delivered outside of their catchment area, and were influenced by the state of their HFCA’s MWH and/or that of a neighboring HFCA.

It is important to note that our data only indicate an association between quality of MWH and utilization of facilities for delivery. We do not know if an MWH is scored as higher quality because it was improved due to an increase of facility deliveries at that site, or if the volume of deliveries changed as a result of the introduction of or improved quality of an MWH. It is also possible that there were substantial changes to the condition of the MWH and/or the facility between the time period for which we captured data on women’s deliveries. It is possible that when the MWH were assessed in 2013, they had deteriorated significantly since 2011, and therefore we are overestimating the rate of facility delivery at low-quality shelters that had previously been medium or high quality shelters. However, this would bias results towards the null and therefore our estimates are likely to be conservative. Another study (Henry et al., under review) suggests that there was very little change in the health facilities’ capacity for EmONC between 2011 and 2013 in Kalomo District during SMGL implementation. We also controlled for SMGL implementation, which would have likely accounted for any improvements at the facilities and the MWH in Kalomo during that time, as well as significant investments in the training and support of Safe Motherhood Actions Groups (SMAGs), community health volunteers who promote safe delivery and birth practices at the household level.

There are additional deterrents to MWH utilization that we were not able to capture in our study, including an increase in costs of delivery at a facility compared to delivery at home in MWHs, lack of privacy, and lack of respect from health staff [16]. Other factors may be operating in women’s decision making about utilizing facilities for delivery, such as attitudes, personal norms, and behavioral control [7, 36]. The lack of either provision of food or help with cooking and the limited availability of water and firewood for cooking, were among other factors related to quality that affected a woman’s staying in an MWH in both the global review of MWH utilization as well as the study in Zambia [16, 18]. We were not able to systematically assess these factors in our study, though they may also have had an influence. It also not clear whether or not there are unmeasurable characteristics about the communities that have MWHs that contribute to women’s choosing to deliver at a facility, such as a supportive community environment for maternal health, community outreach activities independent of SMGL, or community chiefs and leadership promoting facility delivery more than in other areas.

Lastly, the generalizability of the findings may be limited, as data were collected from only two districts in Southern Province. Findings are perhaps most generalizable to other primarily rural districts that are culturally or geographically similar to the two study sites.

## Conclusions

Our study provides evidence to suggest that MWHs could be a feasible option to increasing access to facility-based delivery in this context by addressing some of the factors leading to the first and second delays in seeking and reaching maternity care, and that implementation of MWHs in Zambia should pay close attention to community-accepted quality measures. However, it is important to note that MWHs are a link between women in communities and the formal health system, and that MWHs alone would not be sufficient to improve health outcomes for mothers and babies. Other components critical to maternal and newborn care must also be available within the health system including: a definition of risk factors and selection criteria for mothers; a system for identification and referral of the woman; availability of basic and comprehensive EmONC services and providers, and community support [14]. Constructing even high quality MWHs without consideration of these other factors could create demand for quality maternity services without adequate supply, exacerbating factors that lead to the third delay. It will be important to assess changes in service utilization and maternal and newborn health outcomes as further anticipated investments are made into the construction and implementation of MWH throughout Zambia.

## Abbreviations

ANC: Antenatal care
EmONC: Emergency obstetric and newborn care
HC: Health center
HFA: Health facility assessment
IQR: Interquartile range
MWH: Maternity waiting home
SD: Standard deviation
SMAG: Safe Motherhood action Groups
SMGL: Saving Mothers, Giving Life
WHO: World Health Organization
ZamCAT: Zambia chlorhexidine application trial
